# ON THE CHOICE OF HEALTH INEQUALITY MEASURE FOR THE LONGITUDINAL ANALYSIS OF INCOME-RELATED HEALTH INEQUALITIES

**DOI:** 10.1002/hec.2803

**Published:** 2012-02-27

**Authors:** Paul Allanson, Dennis Petrie

**Affiliations:** Economics Studies, University of DundeeDundee, UK

**Keywords:** income-related health inequality, mobility analysis, vertical equity judgements, inequality equivalence criterion, Great Britain

## Abstract

Changes in rank-dependent income-related health inequality measures over time may usefully be decomposed into contributions due to changes in health outcomes and changes in individuals' positions in the income distribution. This paper establishes the normative implications of this type of decomposition by embedding it within a broader analysis of changes in the ‘health achievement’ index. We further show that the choice of health inequality measure implies a particular vertical equity judgement, which may be expressed on a common scale in terms of the concentration index of health changes that would be inequality preserving. We illustrate the empirical implications of this choice by reporting results from a longitudinal analysis of changes in income-related health inequality in Great Britain using the concentration, the Erreygers and Wagstaff indices of health attainments and the concentration index of health shortfalls. Copyright © 2012 John Wiley & Sons, Ltd.

## 1. INTRODUCTION

In a recent paper, Allanson *et al*. (2010; hereafter AGP) considered the characterisation and the measurement of income-related health inequality (IRHI) using longitudinal data. In particular, they provided a decomposition of the change in the concentration index (CI) between two periods into the following: (i) an income-related health mobility index that reveals whether the pattern of individual health changes favours the initially rich or poor and (ii) a health-related income mobility index that captures the effect of income re-ranking, that is, changes in individuals' positions in the income distribution, on cross-sectional IRHI. This paper extends this work in two directions.

First, we explore the normative basis of the AGP decomposition of the change in IRHI by embedding it within a broader analysis of the change in the health of the population. Specifically, we note that the CI is the inequality component of the ‘health achievement’ index of Wagstaff (2002). Accordingly, any change in health achievement can be broken down into parts due to changes in mean health and changes in IRHI, with the AGP mobility indices identifying the extent to which changes in IRHI are driven by changes in health outcomes (i.e. ‘health mobility’) and income re-ranking (i.e. ‘income mobility’).

Second, we show that the AGP decomposition procedure may also be used to analyse the change in other rank-dependent IRHI indices and with IRHI measured with respect to health shortfalls rather than attainments. The choice of health inequality measure can affect the ranking of populations according to IRHI in comparative studies, and this sensitivity will inevitably carry over to mobility indices derived from the decomposition of changes in IRHI measures over time. Specifically, Clarke *et al*. (2002) showed that the use of relative and absolute indices can produce different rankings and, moreover, that whether health is measured with respect to health attainments or shortfalls also matters in the case of relative indices. The sensitivity of relative measures has led to a search for the class of indices that yield either identical or consistent findings irrespective of the choice between health attainments and shortfalls (Erreygers, 2009; Lambert and Zheng, 2011). However, although health attainments and shortfalls are but ‘two sides of the same coin’ (Clarke *et al*., 2002), indices of inequality measured with respect to health attainments and shortfalls need not be equivalent to each other. In particular, this paper establishes the nature of the distinct vertical equity judgements^1^ embodied in relative indices of inequality in health attainments and shortfalls, which shows that each may represent ethically defensible positions in specific contexts.

To consider the normative implications of the choice of IRHI measure, we build on the observation that different measures embody different IRHI equivalence criteria reflecting alternative vertical equity judgements.^2^ An IRHI equivalence criterion specifies how, given the joint distribution of health and income, an additional amount of health should be distributed so as to leave IRHI unchanged. For example, relative and absolute indices are invariant to equiproportionate and uniform changes in health, respectively. But, in contrast to absolute indices, relative indices imply different inequality equivalence criteria depending on whether health is measured with respect to attainments or shortfalls because an equiproportionate change in attainments will not generally constitute an equiproportionate change in shortfalls and *vice versa*. In this paper, we characterise the IRHI equivalence criteria on a common scale by expressing them in terms of the CI of health changes that would be inequality preserving or distributionally neutral. For each health inequality measure, this provides a comparable benchmark against which to quantify the degree to which the actual distribution of health changes deviates from distributional neutrality and therefore results in a change in measured health inequality.^3^

In keeping with the income inequality literature (see, e.g. Lambert, 2001), we label the vertical equity judgements implied by the IRHI equivalence criteria of absolute and relative measures of inequality in health attainments as ‘leftist’ and ‘rightist’, respectively. These labels were introduced by Kolm (1976) on the basis that a uniform increase in income levels is commonly perceived as more egalitarian than an equiproportionate rise, although the identification of these positions with the political spectrum is problematic in that an equiproportionate reduction in incomes (e.g. due to the imposition of a tax) is generally viewed as more progressive than a uniform decrease (Atkinson, 1983). Our usage of the labels reflects the fact that health attainment is a ‘good’ like income, and it is similarly apparent in this context that whether ‘leftist’ views would be considered more egalitarian than ‘rightist’ views will depend on whether one is evaluating positive or negative health changes—an egalitarian might well prefer uniform health improvements but equiproportionate health depreciation.

We follow Zheng (2007) in further identifying ‘intermediate’ IRHI measures that increase as a result of equiproportionate improvements in health across income classes but fall in response to uniform improvements, ‘extreme rightist’ measures that fall as a result of equiproportionate health improvements and ‘extreme leftist’ measures that increase in response to uniform improvements. We proceed to demonstrate that relative measures of inequality in health shortfalls reflect an ‘extreme leftist’ view, in contrast to the ‘rightist’ view embodied in relative measures of inequality in health attainment.

The following section establishes the normative implications of the AGP type of decomposition by embedding it within a broader analysis of changes in ‘health achievement’. Sections 3 and 4 show that the AGP decomposition procedure may also be used to analyse the change in other rank-dependent IRHI indices and with IRHI measured with respect to health shortfalls rather than attainments. We thus demonstrate that the choice of health inequality measure implies a particular vertical equity judgement, which may be expressed on a common scale in terms of the CI of health changes that would be inequality preserving. Section 5 illustrates the implications of this choice for the longitudinal analysis of IRHI in Great Britain over the 5-year period 1999 to 2004 using a measure of quality-adjusted life years (QALYs) derived from the SF-6D instrument (Brazier *et al*. 2002).

## 2. HEALTH ACHIEVEMENT AND THE LONGITUDINAL ANALYSIS OF HEALTH INEQUALITIES

We draw upon the literature on the ethical foundations of health inequality measurement to elucidate the normative basis of the mobility indices resulting from the longitudinal analysis of IRHI. In particular, we show how the decomposition of changes in IRHI can be embedded within a broader analysis of changes in population health as measured by the ‘health achievement’ index of Wagstaff (2002).

Our analysis focuses on a single transition between an initial period *s* and some final period *f* (*f* > *s*). Let *h_it_* and *R_it_* denote, respectively, the health attainment and the fractional income rank of individual *i* (*i* = 1, …, *n*) in period *t* (*t* = *s*, *f* ), where the health measure lies in the bounded interval *a* ≤ *h_it_* ≤ *b* with *a* ≥ 0 by assumption. Following Wagstaff (2002),^4^ the level of health achievement in any period is defined as the weighted average health of all individuals, where the weights are determined by individuals' ranks in the income distribution. Specifically, let H*_tt_* denote ‘health achievement’ evaluated on the basis of health outcomes in period *t* and fractional income ranks in period *t*,


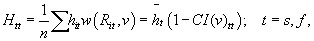
(1)

where the rank-dependent weights are given by


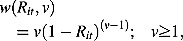
(2)


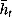
 is the average health attainment and 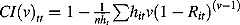 is the extended CI (cf. Yitzhaki, 1983; Wagstaff, 2002). *H_tt_* may be interpreted as a Yaari-type (1988) social evaluation function defined over the health domain, where the ‘distributional judgement’ parameter *v* allows for the calibration of the poverty focus of the evaluation (Essama-Nssah, 2005) by controlling the rate at which the weights decrease from poorest to richest (Wagstaff, 2002). Throughout the remainder of this paper, we implicitly assume that *v* = 2, which yields the (ordinary) CI *CI_tt_*.^5^ *H_tt_* cannot be considered as a full social welfare function as it does not allow for the direct effect of the level of income on welfare.^6^

*CI_tt_* provides a measure of relative IRHI, which typically will be positive because of the positive association between income and health attainment. Within the framework provided by Equation (1), *CI_tt_* may be interpreted as a ‘cost of inequality’ index in the sense of Atkinson (1970), providing a measure of the proportion of average health attainment that could be sacrificed with no loss of ‘health achievement’ if the remainder were to be distributed equally. Bleichrodt and van Doorslaer (2006) have shown that the CI implies social preferences over health distributions must satisfy *inter alia* the principle of income-related health transfers whereby a positive health transfer from a richer to a poorer individual does not lead to a reduction in ‘health achievement’, holding the income rankings of the two individuals constant. If health attainment is an increasing function of income then such transfers will on average be from healthier to unhealthier individuals, but this might not be so in particular cases. Bleichrodt and van Doorslaer (2006, p. 955) concluded that the principle will ‘be more acceptable the stronger the correlation between health and […] income’.^7^

### 2.1. Allanson–Gerdtham–Petrie analysis of changes in relative inequality in health attainments

Allanson *et al*. (2010) proposed a decomposition of the change in the CI between two periods into income-related health and health-related income mobility indices. The key to this decomposition is the definition of the level of IRHI that would result if health outcomes in period *f* were evaluated on the basis of income ranks in period *s*,


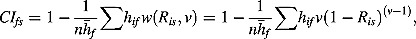
(3)

where *CI_fs_* is interpreted as the CI of final period health ranked by initial income. Hence, the change in overall ‘health achievement’ between the two periods can be written as


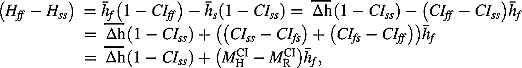
(4)

where 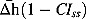 gives the effect on ‘health achievement’ of the mean change in health attainment 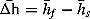 and 
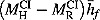
 results from the change in relative inequality (*CI_ff_* − *CI_ss_*). Hence, increases in inequality in health attainment will reduce ‘health achievement’ and *vice versa*, with the AGP decomposition identifying whether the changes in IRHI are driven by changes in health outcomes or by income re-ranking.

The income-related health mobility index 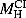 in Equation (4) captures the effect of health changes on relative IRHI, which is determined by the relationship between relative health changes and initial income ranks,


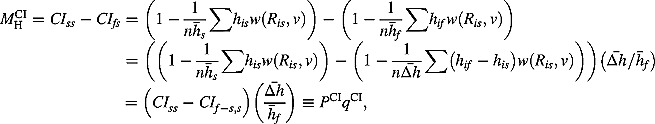
(5)

where *CI*_*f* − *s*,*s*_ is the CI of health attainment changes ranked by initial period income. Hence, AGP evaluated health mobility on the basis of the social weights associated with individuals' initial income ranks, which gives greater weight to the health prospects of those who start with lower income. Following Dardanoni (1993), this asymmetric treatment of individuals can be justified on the grounds that the initially poor are disadvantaged to the extent that they face a worse lottery of future health possibilities than those who are better off. 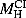 will be positive if the prospect of health mobility is deemed socially desirable in the sense that the resulting distribution of health is less unequal than the initial health distribution, when both are judged on the basis of the social weights from the initial period.

Progressivity is captured by the Kakwani-type (1977) progressivity index *P*^CI^ = (*CI_ss_* − *CI*_*f* − *s*,*s*_). *P*^CI^ will be positive (negative) if the poorest individuals either enjoy a larger (smaller) share of total net health gains or suffer a larger (smaller) share of total net health losses compared with their initial share of health attainment and equals 0 if relative health changes are independent of income. So, for example, positive values of *P*^CI^ imply that health changes will be equalising for net health improvements and disequalising for net health deteriorations. For any given *P*^CI^, the gross impact of health mobility on IRHI is proportional to the scale of health changes 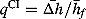. *P*^CI^ can provide a useful measure of the performance of health improvement programmes in targeting the poor: A given reduction in IRHI can be achieved either by a small-scale but highly targeted intervention or by a larger-scale but broader health programme.

The health-related income mobility index 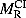 in Equation (4) captures the effect of income re-ranking on (relative) IRHI, which is determined by the relationship between individuals' final health level and income rank changes:


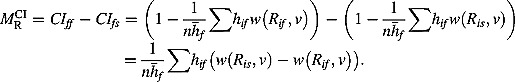
(6)


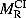
 will be positive, exacerbating IRHI, if *CI_ff_* is greater than *CI_fs_*, which will be the case if current health is more strongly related to contemporaneous than lagged income ranks.^8^ AGP argued that 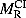 may generally be expected to be positive because those who move up the income ranking will tend to be healthier in the final period than those who move down.

## 3. LONGITUDINAL ANALYSIS OF CHANGES IN OTHER RANK-DEPENDENT INCOME-RELATED HEALTH INEQUALITY INDICES OF HEALTH ATTAINMENT

Allanson *et al*. (2010) considered the decomposition of the change in the CI of health attainments, but the same approach may also be applied to a number of alternative rank-dependent IRHI indices that have appeared in the literature: the generalised concentration index (GC), relative and slope inequality indices (RII and SII respectively), and the Wagstaff (2005) index (WI) and Erreygers (2009) index (EI) that ‘normalise’ or ‘correct’ the CI to take account of the bounded nature of the health measure. Table I provides definitions for all the IRHI indices considered in the study with respect to health attainments, together with the corresponding sets of mobility indices.

**Table I:** Definition of income-related health attainment inequality measures and associated mobility indices

	Definition	Income-related health mobility	Progressivity index	Scale factor	Health-related income mobility
*‘Rightist’ inequality equivalence criterion*					
Concentration index	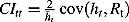	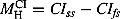	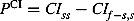	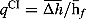	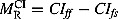
Relative inequality index	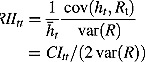	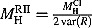	*P*^RII^ = *P*^CI^	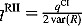	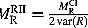
*‘Leftist’ inequality equivalence criterion*					
Generalised concentration index	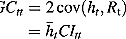	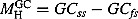	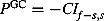	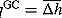	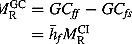
Slope inequality index	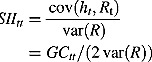	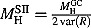	*P*^SII^ = *P*^GC^	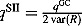	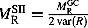
Erreygers (2009) index	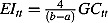	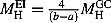	*P*^EI^ = *P*^GC^	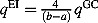	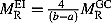
*‘Variable’ inequality equivalence criterion*					
Wagstaff (2005) index	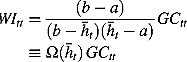	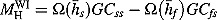	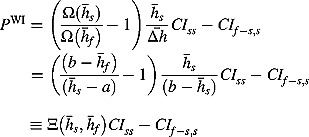	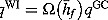	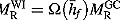

See Section 4 for comparable definitions if health is measured with respect to shortfalls rather than attainments and a discussion of the normative implications of this change. The variance of the relative rank variable, var(*R*), depends only on sample size and is therefore invariant over time. Generalised concentration index and slope inequality index measures are in the same units as the health attainment measure. All other inequality indices are dimensionless.

The derivation of the income-related health and health-related income mobility indices, *M*_H_ and *M*_R_, respectively, is straightforward for these other IRHI indices.^9^ However, the appropriate definition of the progressivity and scale indices, *P* and *q*, respectively, is less obvious: We define *P* such that differences between alternative progressivity indices can be interpreted as reflecting differences between the IRHI equivalence criteria implied by the underlying health inequality measures, with *q* defined conformably such that *M*_H_ = *Pq*.

To this end, we characterise the IRHI equivalence criterion implied by each IRHI index in terms of the CI of health changes that would be inequality preserving or distributionally neutral, where each such benchmark may be expressed as some multiple *θ* of the CI of initial health *CI_ss_*. To quantify the degree to which the actual distribution of health changes deviates from distributional neutrality, we then define *P* in each case as the difference between the benchmark function *θCI_ss_* and the actual CI of health changes *CI*_*f* − *s*,*s*_.

*P* is a dimensionless measure that provides a generalisation of the progressivity index *P*^CI^ in Equation (5). For all IRHI measures that have the same IRHI equivalence criterion, *P* will be identical, reflecting the common vertical equity judgement, whereas the scale indices *q* will differ because of the alternative normalisations. More generally, the ratio of *P* values between any two IRHI measures will be equal to the ratio of income-related health mobilities if these are expressed as proportions of the corresponding final health inequalities.^10^

### 3.1. Income-related health inequality measures embodying a ‘rightist’ inequality equivalence criterion

The decomposition results for the CI were discussed in the previous section. In this case, *θ* = 1, and the benchmark function *θCI_ss_* = *CI_ss_* implies that an equiproportionate change in health will be deemed to be inequality preserving. The RII is commonly used instead of CI to measure relative inequality and is similarly invariant to equiproportionate changes. Thus, *P*^RII^ = *P*^CI^, reflecting the common ‘rightist’ vertical equity judgement.

Wagstaff *et al*. (1991) noted that the RII is equal to the CI divided by twice the variance of the relative rank variable, where var(*R*) = (*n* − 1)^2^/(12*n*^2^) is a constant determined by the sample size *n* (Milanovic, 1997). Thus, the two measures and the resultant mobility and scale indices simply differ by a multiplicative factor, with the RII values being approximately six times the corresponding CI values in large samples.

### 3.2. Income-related health inequality measures embodying a ‘leftist’ inequality equivalence criterion

The GC is an absolute measure of inequality that is invariant to uniform health changes, so *θ* = 0, and the benchmark function is simply given as *θCI_ss_* = 0. 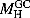 may thus be expressed as the product of the progressivity index *P*^GC^ = −*CI*_*f* − *s*,*s*_, which provides an alternative ‘leftist’ indicator of targeting performance to *P*^CI^, and a scale factor 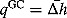. The belief that the priority of healthcare policy should be to ‘heal the sick’, who are disproportionately poor, may be taken to imply that healthcare improvements should be equalising not just in relative but also in absolute terms,^11^ that is, that *P^GC^* should be positive for beneficial health interventions.^12^ Conversely, a uniform loss in health may be seen as less egalitarian than an equiproportionate one given that the poor typically start with worse health.

The SII and EI are similarly invariant to uniform health changes. Thus, *P*^GC^ = *P*^SII^ = *P*^EI^, reflecting the common ‘leftist’ vertical equity judgement. The SII provides an absolute IRHI index equal to the RII multiplied by mean health, so the set of SII and GC mobility and scale indices differ by the same multiplicative factor as the corresponding RII and CI indices. The EI provides a dimensionless IRHI index that is equal to (4/(*b* − *a*))*GC*, so the EI mobility and scale indices are equal to (4/(*b* − *a*)) times the corresponding GC indices.

### 3.3. Income-related health inequality measure embodying a ‘variable’ inequality equivalence criterion

Finally, we note that the WI does not embody some fixed vertical equity judgement, as is the case with rightist and leftist measures, because 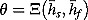 implies that the benchmark function 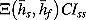 depends upon the scale of the average health change as well as the initial distribution of health.^13^ Note first that if 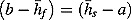, which will be the case if average health in each period is equal to the midpoint of the range,^14^ then WI embodies a ‘leftist’ vertical equity judgement because 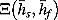 will be equal to 0. For low average levels of both initial and final health, 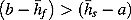, so 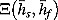 will be positive, tending to 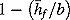 as average initial health tends to *a* if *a* = 0 and to positive infinity if *a* > 0. Thus, in this case, WI will provide an ‘intermediate’ measure if *a* = 0 and tend to an ‘extreme rightist’ one otherwise. Conversely, for high initial and final average health 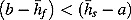, so WI will provide an ‘extreme leftist’ measure with 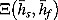 tending to negative infinity as average initial health tends to *b*.^15^

## 4. LONGITUDINAL ANALYSIS OF CHANGES IN INCOME-RELATED HEALTH INEQUALITY IN HEALTH SHORTFALLS

Thus far, we have focused on income-related inequalities in health attainment, but the preceding analysis may also be applied to changes in income-related inequalities in health shortfalls or morbidity. For this purpose, let *u* = (*b* − *h*) be some measure of health shortfalls,^16^ with corresponding bounds 0 ≤ *u* ≤ (*b* − *a*). We first show that the conclusions drawn from the longitudinal analysis of IRHI will be sensitive to the choice between attainments and shortfalls if IRHI is evaluated in relative terms because the two approaches embody fundamentally different vertical equity judgements. We subsequently note that this is not an issue if the IRHI index satisfies the ‘mirror’ condition—that health inequalities measured with respect to attainments and shortfalls are of equal size but of opposite sign—which is the case for all the other IRHI indices considered in this paper (Erreygers, 2009).

Taking the CI as an example of a relative inequality index, ‘health achievement’ may be re-written from Equation (1) as 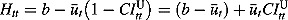, where *ū*_*t*_ is the average health shortfall and 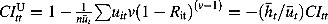 is the CI of health shortfalls. 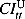, unlike 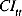, will typically be negative, reflecting the concentration of shortfalls among the poor, with such inequalities in shortfalls leading to a loss in ‘health achievement’.

Hence, the change in ‘health achievement’ in Equation (4) can be re-written as


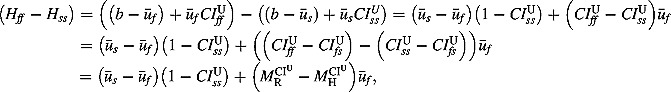
(7)

where 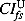 is the CI of final period health shortfalls ranked by initial income. Thus, reductions in both average health shortfalls and (the scale of) relative IRHI in shortfalls will improve overall well-being, with the AGP decomposition of 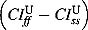 serving to identify the sources of the latter as before.

The income-related health mobility index 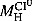 will be positive if expected changes in health shortfalls conditional upon income rank have the effect of increasing IRHI in shortfalls. 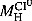 may be expressed as the product of the progressivity of health shortfall changes 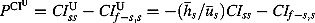, where 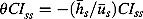 is the benchmark function and 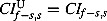 by definition, and the scale of such changes 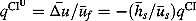. 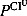
will be positive (negative) if changes in health shortfalls are more (less) concentrated among the poor than the initial concentration of shortfalls and will equal 0 if relative changes in shortfalls are independent of income. So, for example, positive values of 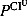 imply that health changes will be equalising in shortfalls for net reductions in shortfalls (i.e. health improvements) and disequalising for net increases in shortfalls (i.e. health deteriorations).


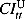
 can thus be identified as an ‘extreme leftist’ measure of IRHI because the benchmark *θCI_ss_* < 0 if, as usually will be the case, *CI_ss_* is positive, implying that a uniform health improvement will increase IRHI according to this measure.^17^ Accordingly, 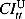 provides a benchmark of distributional neutrality for the evaluation of public health interventions that is consistent with the principle of ‘proportionate universalism’ advocated in the Marmot Review (Marmot, 2010, p. 15): ‘To reduce the steepness of the social gradient in health, actions must be universal, but with a scale and intensity that is proportionate to the level of disadvantage’. To be precise, both relative and absolute IRHI in health attainments will be reduced by proportionate universal interventions that result in equiproportionate reductions in the burden of health shortfalls across income classes because 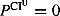 implies both 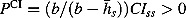 and 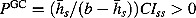 if *CI_ss_* is positive.

The health-related income mobility index 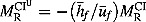 will be negative if, as expected, 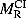 is positive, implying that those who move up the income ranking will tend to be less unhealthy in the final period than those who move down. Thus, re-ranking will generally exacerbate inequalities in health shortfalls as well as in health attainment.

In contrast to relative IRHI indices, the results of the decomposition analysis will be insensitive to the choice between health attainments and shortfalls for any IRHI index that satisfies the ‘mirror’ condition. Specifically, let *JJ* (*JJ* = *GC*, *SII*, *EI*, *WI*) be one such index, then 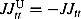 by definition, 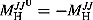, with 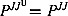 and 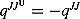, and 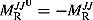. Thus, the signs of the mobility and scale indices change, but the sign of the progressivity index does not as the vertical equity judgement is the same irrespective of the choice of health measure.

## 5. EMPIRICAL ILLUSTRATION

In the preceding sections, we have shown how to decompose changes in IRHI for a number of commonly used measures and thereby revealed that between them they embody only four distinct vertical equity judgements. We illustrate the empirical implications of these four vertical equity judgements by reporting results from a study of the dynamics of income and health in Great Britain for the CI, EI and WI of health attainments and for the CI of health shortfalls. Results for the other IRHI measures considered in the paper can readily be derived using the mathematical relationships between the various measures set out earlier.

The empirical analysis employs data from waves 9 and 14 of the British Household Panel Survey (University of Essex, ISER, 2007), which is a nationally representative longitudinal survey of private households in Great Britain, to construct a balanced panel consisting of observations on the sub-set of individuals in the survey who were alive in 1999 and 2004 and for whom full information on health, income and a range of other socioeconomic variables was available in both years. The resultant sample comprises observations on 9258 individuals in Great Britain. Sample weights were used throughout the analysis, with these being given by a set of adjusted British Household Panel Survey cross-sectional weights for 1999, where the adjustments were made using inverse probability weights (Wooldridge, 2002) to allow both for missing data in either year and for non-mortality-related sample attrition between the years (see Petrie *et al*., 2011, for further discussion). Standard errors were generated for all estimates using a bootstrap procedure that took into account the sample design, with re-sampling carried out at the cluster level (primary sampling unit) rather than at the individual level within each major strata.^18^

We employ a measure of health attainment that is expressed in terms of QALYs and derived from responses to the SF-36 questionnaire using the SF-6D preference-based algorithm (Brazier *et al*., 2002). This measure is bounded in the unit interval with full health corresponding to a value of 1 and death assigned a score of 0,^19^ so our measure of health shortfalls is simply equal to one less the measure of health attainment. We rank individuals on the basis of annual household income, equivalised using the McClements scale (Taylor, 1995) to take account of household composition.

Table II shows that average health declined over the 5-year period, which is to be expected given the balanced nature of the panel. Health attainments were concentrated among the rich and health shortfalls among the poor in 1999, as is indicated by the signs of the corresponding measures of health inequality. The benchmark function value for each measure gives the CI of health changes that would preserve IRHI. For the two relative measures, *CI* and *CI* ^u^, the benchmark values are identical to the corresponding levels of initial inequality, given that equiproportionate health changes are inequality preserving in these cases. But note that such health changes will be concentrated among the rich in the case of the ‘rightist’ *CI* and among the poor for the ‘extreme leftist’ *CI* ^u^. The benchmark value for the ‘leftist’ *EI* is equal to 0 by definition, given that uniform health changes would be inequality preserving. Finally, given the high levels of average health in both 1999 and 2004, the *WI* benchmark value implies that health changes would have to be concentrated among the poor to be inequality preserving, making *WI* an ‘extreme leftist’ measure but less so than *CI* ^u^.

**Table II:** Decomposition of changes in income-related health inequalities

		Health attainments		Health shortfalls
Average initial health outcome		0.8091 *(0.0017)*		0.1909 *(0.0017)*
Average health outcome change		−0.0096 *(0.0016)*		0.0096 *(0.0016)*
Concentration index of health changes		−0.3179 *(0.1162)*		−0.3179 *(0.1162)*

	Concentration index *CI*	Erreygers index *EI*	Wagstaff index *WI*	Concentration index *CI^u^*
Initial inequality	0.0167 *(0.0011)*	0.0541 *(0.0036)*	0.0876 *(0.0058)*	−0.0709 *(0.0047)*
Benchmark function value	0.0167 *(0.0011)*	0 *(−)*	−0.0533 *(0.0035)*	−0.0709 *(0.0047)*
Change in inequality	0.0084 *(0.0012)*	0.0262 *(0.0039)*	0.0377 *(0.0062)*	−0.0293 *(0.0050)*
Income-related health mobility	−0.0040 *(0.0011)*	−0.0122 *(0.0036)*	−0.0158 *(0.0057)*	0.0118 *(0.0046)*
Progressivity index	0.3347 *(0.1158)*	0.3179 *(0.1162)*	0.2646 *(0.1177)*	0.2471 *(0.1183)*
Scale factor	−0.0119 *(0.0020)*	−0.0382 *(0.0064)*	−0.0596 *(0.0097)*	0.0506 *(0.0086)*
Health-related income mobility	0.0044 *(0.0010)*	0.0141 *(0.0031)*	0.0220 *(0.0048)*	−0.0176 *(0.0038)*
Final inequality	0.0251 *(0.0013)*	0.0803 *(0.0039)*	0.1253 *(0.0059)*	−0.1002 *(0.0046)*

The benchmark function value for each measure gives the concentration index of health changes that would preserve income-related health inequality. Bootstrap standard errors based on 2000 replications are in parentheses.

Health inequality rose over the 5-year period according to all the IRHI measures considered in the study, which is evident from the larger magnitudes of all the reported inequality values in 2004. The subsequent decomposition results show how much of the rise in IRHI in each case was driven by changes in health outcomes and how much by income re-ranking.

Income-related health mobility had a disequalising effect in this study, irrespective of the choice of health inequality measure, due to the concentration of health losses among the poor being such that the CI of health changes was more negative than any of the benchmark values. Thus, the progressivity index is positive in each case, with the smallest deviation from neutrality given by the ‘extreme leftist’ *CI* ^u^, the largest by the ‘rightist’ *CI*, and with *WI* and *EI* in between. Income-related health mobility, expressed as a proportion of final health inequality, was 11.5% in the case of *CI* ^u^ and 15.9% for *CI*, with the latter value being 35.3% larger than the former where this ratio is also given by the ratio of the corresponding *P* values.

Although in this example health mobility was disequalising according to all the measures, this need not be the case. If the value of the CI of health changes had fallen between a pair of benchmark values, then one measure would have implied that health mobility was equalising whereas the other would have suggested it was disequalising. For example, a uniform health loss across all income classes, for which *CI*_*f* − *s*,*s*_ = 0, would have had an equalising effect according to *CI* ^u^ and *WI*, been inequality preserving according to *EI* and been disequalising according to *CI*. And if the actual CI of health changes had been more positive than any benchmark value, then health losses would have been equalising according to all measures.

In contrast to income-related health mobility, health-related income mobility will always be either equalising or disequalising irrespective of the choice of health inequality measure. Indeed, health-related income mobility, expressed as a proportion of final IRHI, must be the same across all IRHI measures. In this study, those who moved up the income ranking tended to be healthier in 2004 than those who moved down, such that 17.5% of IRHI in 2004 was due to re-ranking. Thus, income re-ranking had more of a disequalising effect than health changes for all the IRHI measures. Overall, between 29.0% and 33.4% of IRHI in 2004 had arisen because of the combination of health and income rank changes since 1999.

## 6. CONCLUSIONS

This paper elaborates the approach to the longitudinal analysis of IRHI proposed by Allanson *et al*. (2010). The resultant contribution is twofold. First, we establish the normative basis of the AGP mobility indices by embedding the AGP decomposition of changes in IRHI within a broader analysis of changes in ‘health achievement’. In particular, we show that the income-related health mobility index provides a forward-looking measure in which the redistributive effect of individuals' health changes are evaluated on the basis of their initial income ranks, with this asymmetric treatment justifiable on the grounds that the initially poor are disadvantaged to the extent that they face a worse lottery of future health possibilities than the better off. We further show that income re-ranking will lead to a loss of ‘health achievement’ to the extent that it exacerbates IRHI.

Second, we demonstrate that the AGP decomposition procedure may be used to analyse changes in rank-dependent IRHI measures other than the CI. The choice of health inequality measure is shown to affect the results of the subsequent longitudinal analysis only to the extent that different measures embody alternative inequality equivalence criteria. In particular, relative measures of inequality in health attainments and in health shortfalls embody fundamentally different vertical equity judgements that may affect whether changes in health outcomes are perceived as equalising or disequalising. In contrast, inequality indices that satisfy the ‘mirror’ condition, such as GC, WI and EI, have the practical property that the conclusions of any longitudinal analysis will be invariant to whether IRHI is measured with respect to health attainments or shortfalls. However, the exact choice of health inequality measure should not be guided by analytical convenience but by perceptions of what distribution of health changes between poor and rich would constitute an improvement in IRHI in a particular context, where this may well depend on whether one is evaluating positive or negative health changes. Studies should therefore justify the vertical equity judgements implied by their choice of health inequality measure or present findings for a range of measures to provide policymakers with a more detailed assessment of the nature of the health inequality changes taking place.

## Footnotes

1 See Culyer and Wagstaff (1993) for a discussion of equity issues in health and health care.

2 See Zoli (2003) for a discussion of inequality equivalence criteria with reference to income inequality.

3 Note that this approach to assessing the nature of health inequality changes over time could also be applied when comparing health inequality differences across populations.

4 Wagstaff considers an indicator that provides a measure of ill health, or health shortfall, rather than health attainment. We examine how this affects the analysis in Section 4.

5 The analytical results in this paper hold for all admissible values of *v*.

6 We are grateful to an anonymous referee for pointing this out. See Weymark (2006) for a discussion of multidimensional welfare measurement.

7 We note that *CI_tt_* is unaffected by the degree of conditional dispersion of health outcomes because all individuals with the same income will be given the same weight *w*(*R_it_*, *v*) in the determination of ‘health achievement’.

8 It is readily shown that *CI_ff_* > *CI_fs_* implies corr(*h_f_*,*R_f_*) > corr(*h_f_*,*R_s_*) in the special case *v* = 2 (Milanovic, 1997).

9 It is also possible to embed these decompositions of the change in inequality into broader analyses of the change in health achievement. In particular,  for the GC.

10 For example, .

11 Dolan *et al*. (2005) reported evidence, obtained from a review of relevant studies, to the effect that people are generally in favour of giving priority to those who are most severely ill, concluding that the marginal social value of health improvements is diminishing in initial health.

12 *P*^GC^ > 0 implies *P*^CI^ > 0 if *CI_ss_* > 0 because the poor must experience a larger share of total health gains than their initial share of health if absolute health gains are concentrated among the poor, but *P*^CI^ > 0 does not necessarily imply *P*^GC^ > 0.

13 See Zoli (2003) for further discussion of the nature of inequality equivalence criteria.

14 More generally, the condition will hold if average health over the two periods is equal to the midpoint of the range.

15 Erreygers and van Ourti (2011) provided a similar exploration of the properties of the Wagstaff measure in terms of the elasticity of the ‘normalisation function’ of the index  with respect to mean health , using the labels ‘inverse relative’, ‘mixed’ and ‘inverse absolute’ for what we refer to as ‘extreme leftist’, ‘intermediate’ and ‘extreme rightist’ measures, respectively. Kjellsson and Gerdtham (2011) introduced the notion of ‘mirror relativity’ to describe the vertical equity judgements embodied in the WI, noting that the measure suggests that the same level of absolute inequalities is more severe when the level of health attainment is either high or low. See also Wagstaff (2009).

16 The definitions of *h* and *u* may be reversed if one has a primitive indicator of health status that provides a measure of morbidity with a non-zero origin. This section would then refer to the derived measure of health attainment.

17 This follows because  will be positive in this case as  given that  by definition for a uniform health change and  given that a health improvement reduces health shortfalls. We further note that  by definition, such that  always embodies a more ‘extreme leftist’ judgment than .

18 However, the bootstrapping procedure did not include re-estimation of the individual weights that were constructed from the original sample. See Biewen (2002) on the use of bootstrap inference for inequality and mobility measurement.

19 Note that the lowest QALY possible for those alive is 0.308, which could alternatively be used as the lower bound in the EI and WI measures instead of 0.
